# Tafenoquine treatment of *Plasmodium vivax* malaria: suggestive evidence that CYP2D6 reduced metabolism is not associated with relapse in the Phase 2b DETECTIVE trial

**DOI:** 10.1186/s12936-016-1145-5

**Published:** 2016-02-18

**Authors:** Pamela L. St Jean, Zhengyu Xue, Nick Carter, Gavin C. K. W. Koh, Stephan Duparc, Maxine Taylor, Claire Beaumont, Alejandro Llanos-Cuentas, Ronnatrai Rueangweerayut, Srivicha Krudsood, Justin A. Green, Justin P. Rubio

**Affiliations:** PAREXEL International, 2520 Meridian Parkway, Durham, NC 27713 USA; GlaxoSmithKline, Stockley Park West, Uxbridge, Middlesex UK; Medicines for Malaria Venture, Geneva, Switzerland; GlaxoSmithKline, Ware, Hertfordshire, UK; Instituto de Medicina Tropical, Alexander von Humboldt, Universidad Peruana Cayetano Heredia, Lima, Peru; Mae Sot Hospital, Mae Sot, Tak Province Thailand; Faculty of Tropical Medicine, Mahidol University, Bangkok, Thailand; Department of Pathology, University of Melbourne, Melbourne, VIC Australia

**Keywords:** *Plasmodium vivax* malaria, Tafenoquine, Primaquine, CYP2D6, Efficacy, Pharmacogenetics, Pharmacokinetics

## Abstract

**Background:**

Tafenoquine (TQ) and primaquine (PQ) are 8-aminoquinolines (8-AQ) with anti-hypnozoite activity against vivax malaria. PQ is the only FDA-approved medicine for preventing relapsing *Plasmodium vivax* infection and TQ is currently in phase 3 clinical trials for the same indication. Recent studies have provided evidence that cytochrome P450 (CYP) metabolism via CYP2D6 plays a role in PQ efficacy against *P. vivax* and have suggested that this effect may extend to other 8-AQs, including TQ. Here, a retrospective pharmacogenetic (PGx) investigation was performed to assess the impact of CYP2D6 metabolism on TQ and PQ efficacy in the treatment of *P. vivax* in the DETECTIVE study (TAF112582), a recently completed, randomized, phase 2b dose-ranging clinical trial. The impact of CYP2D6 on TQ pharmacokinetics (PK) was also investigated in TAF112582 TQ-treated subjects and in vitro CYP metabolism of TQ was explored. A limitation of the current study is that TAF112582 was not designed to be well powered for PGx, thus our findings are based on TQ or PQ efficacy in CYP2D6 intermediate metabolizers (IM), as there were insufficient poor metabolizers (PM) to draw any conclusion on the impact of the PM phenotype on efficacy.

**Methods:**

The impact of genetically-predicted CYP2D6 reduced metabolism on relapse-free efficacy six months post-dosing of TQ or PQ, both administered in conjunction with chloroquine (CQ), was assessed using exact statistical methods in 198 *P. vivax*-infected study participants comparing IM to extensive metabolizers (EM). The influence of CYP2D6 metabolizer phenotypes on TQ PK was assessed comparing median TQ area under the curve (AUC). *In vitro* metabolism of TQ was investigated using recombinant, over-expressed human CYP enzymes and human hepatocytes. Metabolite identification experiments were performed using liquid chromatography-mass spectrometry.

**Results:**

Reduction of CYP2D6 activity was not associated with an increase in relapse-rate in TQ-treated subjects (p = 0.57). In contrast, and in accordance with recent literature, CYP2D6 IMs were more common (p = 0.05) in PQ-treated subjects who relapsed (50 %) than in subjects who remained relapse-free (17 %). Further, CYP2D6 metabolizer phenotypes had no significant effect on TQ AUC, and only minimal metabolism of TQ could be detected in hepatic in vitro systems.

**Conclusion:**

Together, these data provide preliminary evidence that in CYP2D6 IMs, TQ efficacy in *P. vivax*-infected individuals is not diminished to the same extent as PQ. As there were no PMs in either the TQ or PQ treatment arms of TAF112582, no conclusions could be drawn on potential differences in PMs. These findings suggest that differential effects of CYP2D6 metabolism on TQ and PQ efficacy could be a differentiation factor between these 8-AQs, but results remain to be confirmed prospectively in the ongoing phase 3 studies.

**Electronic supplementary material:**

The online version of this article (doi:10.1186/s12936-016-1145-5) contains supplementary material, which is available to authorized users.

## Background

Globally, it is estimated that 132–391 million *Plasmodium vivax* clinical infections occur each year [1]. In order to treat and potentially eradicate relapsing forms of malaria, such as *P. vivax*, drugs with anti-hypnozoite activity are required to kill the latent hypnozoite liver-stage of the parasite. Currently, primaquine (PQ) is the only drug approved by the US Food and Drug Administration (FDA) for this indication since schizonticidal drugs such as chloroquine (CQ) do not have anti-relapse efficacy. PQ is a member of the 8-aminoquinoline (8-AQ) drug class, which also includes tafenoquine (TQ). TQ is a synthetic derivative of PQ and is currently in phase 3 clinical development after showing a good efficacy and tolerability profile in a recent phase 2b dose-ranging study [2].

Recent research has highlighted a potential pharmacogenetic (PGx) effect on the efficacy of PQ in individuals who are naturally deficient in cytochrome P450 2D6 (CYP2D6) activity. In both mice and humans, this research has provided consistent evidence that metabolic activation of PQ by CYP2D6 is required for anti-hypnozoite activity, possibly via a toxic metabolite [3–7]. A further study in mice suggested that this CYP2D6 liability may extend to other members of the 8-AQ drug class, including TQ [8]. Most recently, Vuong et al. [9] reported higher TQ exposure [area under the curve (AUC)] in CYP2D knock-out mice compared to wild type and suggested CYP2D6 could potentially affect TQ pharmacokinetics (PK) in humans.

CYP2D6 is a highly polymorphic drug-metabolizing enzyme involved in the biotransformation of numerous medicines and it is well known that genetic polymorphisms in the *CYP2D6* gene influence exposure to many drugs and/or active metabolites and responses [10, 11]. Metabolizer phenotype can be predicted for *CYP2D6* alleles, although underlying metabolic differences between individuals with the same *CYP2D6* genotype can make phenotype inference challenging [10–13]. If, as proposed by Marcsisin et al. [8], the CYP2D6 liability observed for PQ extends to other members of the 8-AQ drug class, the high degree of genetic polymorphism and considerable variability in the distribution of functional alleles across the world, would pose major obstacles to the development of pharmacogenetically guided treatment strategies.

The aim of the current study was to determine whether clinical anti-relapse efficacy of TQ and PQ, as well as TQ PK, are impacted by reduced CYP2D6 activity using a retrospective PGx assessment in subjects from a randomized clinical trial. A complimentary aim was to elucidate potential metabolic effects of CYP enzymes on TQ metabolism in vitro.

## Methods

### Pharmacogenetic study subjects

Participants’ samples were from Part 1 of the seamless Ph2b/3 TAF112582 study, a multi-centred, double-blind, randomized, parallel-group, placebo-controlled study to evaluate the efficacy, safety and tolerability of TQ in subjects infected with *P. vivax* [2]. TAF112582 Part 1 consisted of six treatment arms: CQ (600 mg days 1 and 2, 300 mg day 3) plus TQ and PQ placebos; CQ (doses as above) combined with single dose TQ 50, 100, 300 or 600 mg plus PQ placebo; or CQ (doses as above) combined with PQ 15 mg daily for 14 days plus TQ placebo. Protocol approval was obtained from each site’s ethics committee or institutional review board and prospective written informed consent was obtained for all subjects involved in this PGx study, which was funded by GlaxoSmithKline (GSK) and the Medicines for Malaria Venture (MMV).

### Tafenoquine pharmacokinetics data

A population PK model was developed to characterize systemic TQ concentrations in TAF112582 Part 1 subjects treated with TQ. Model-predicted individual post hoc clearance estimates were utilized to generate the individual exposure (AUC) values for the analyses [14].

### *CYP2D6* genotyping and phenotype inference

Venous blood was collected into an EDTA vacutainer for each of the subjects who consented to PGx research. Genomic DNA was extracted from peripheral blood using the Gentra Puregene kit on the Autopure LS (Qiagen, Valencia, CA, USA) by Quest Diagnostics (Valencia, CA, USA or Heston, UK). *CYP2D6* genotyping was performed by BioProcessing Solutions (Piscataway, NJ, USA) using the Affymetrix^®^ DMET-Plus array (Santa Clara, CA, USA). All genotype calling and quality control was performed in accordance with the manufacturers’ protocols.

Genetically predicted CYP2D6 metabolizer status was determined from this array. The translation of genotype information into metabolizer phenotype is challenging given the range of activity possible for an allele [12] and many classification schemes have been proposed or used in practice [5, 7, 12]. Here, subjects were classified as (1) poor metabolizers (PM) if they carried two ‘null’ (no enzymatic function) alleles; (2) intermediate metabolizers (IM) if they carried one null and one functionally deficient allele, two deficient alleles, or one null allele and one normal (wildtype function) allele; and, (3) extensive metabolizers (EM) if they carried two normal alleles or one normal allele and one deficient allele. In addition, the CYP2D6 ‘activity score’ (AS) [12], which translates genotype into a qualitative measure of enzyme activity, was explored in this study. Briefly, for each study subject, AS was determined by summing the per-allele scores; a null allele having a score of 0, a deficient allele a score of 0.5 and a normal allele a score of 1. Thus, PMs could have an AS of 0, IMs an AS of 0.5 or 1 and EMs an AS of 1.5 or 2.0.

### Statistical analysis

TAF112582 showed poor efficacy for TQ50 and TQ100 mg treatment arms, while higher dose treatment arms, TQ300 and TQ600 mg, showed good efficacy [2]. Therefore, to facilitate interpretation of results, the TQ low dose (50 and 100 mg) and high dose (300 and 600 mg) treatment arms were combined. An exact logistic regression model adjusting for country of origin was used to assess the effect of inferred CYP2D6 metabolizer phenotype or AS on clinical outcome within treatment arm. Because only 1 PM was identified in the clinical study, only subjects with IM or EM phenotypes were included in the analysis. Given published data indicating an effect of CYP2D6 metabolism on PQ efficacy [3–7], a one-sided test was applied to test the one-sided alternative hypothesis that reduced metabolism increased the risk of relapse due to *P. vivax* infection 6 months post-dosing. For the PK assessment, a post hoc analysis using a non-parametric method to compare median TQ AUC between IM and EM subjects was conducted separately in the TQ low dose (50 or 100 mg) and TQ high dose (300 or 600 mg) groups. All analyses were conducted using SAS/STAT (SAS Systemv9.2, SAS Institute Inc, Cary, NC, USA).

### Chemicals in isoenzyme and hepatocyte experiments

Tafenoquine was supplied by Chemical Development, GlaxoSmithKline Research and Development Ltd. (Stevenage, UK). Glucose-6-phosphate (G6P), glucose-6-phosphate dehydrogenase (G6PD) and β-nicotinamide adenine dinucleotide phosphate (NADP) were obtained from Sigma Chemical Company (St Louis, USA). All other commercially obtained chemicals and solvents were of high-performance liquid chromatography (HPLC) or analytical grade.

### Isoenzymes

Supersomes™, containing individually over-expressed human CYP enzymes, derived from baculovirus-infected insect cells, and control Supersomes™ (lacking any native human CYP activity) were obtained from BD Biosciences, Woburn, MA, USA. The enzymes used in this study were: CYP1A2, CYP2C8, CYP2C9, CYP2C19, CYP2D6, CYP3A4 (Lot# 72600, 76739, 73030, 71916, 73755, and 70741, respectively). Supersomes™ expressing CYP2C8, CYP2C9, CYP2C19, and CYP3A4 co-expressed CYP reductase and cytochrome b_5_, while Supersomes™ expressing CYP1A2 and CYP2D6 co-expressed CYP reductase only. TQ was incubated at 5 and 10 μM with Supersomes™ (BD Gentest, Woburn, MA, USA), overexpressing individual cytochrome P450 enzymes 1A2, 2C8, 2C9, 2C19, 2D6, or 3A4 at 300 pmol/ml P450, in phosphate buffer (50 mM, pH 7.4) at 37 °C in a shaking water bath. Following an equilibration period of 5 min at 37 °C, reactions were started by the addition of pre-warmed cofactor solution [a NADPH regenerating system containing 1.7 mg of NADP, 7.8 mg of G6P, and six units of G6PD per ml of 2 % (w/v) sodium hydrogen carbonate]. Incubations were conducted under conditions which limited the exposure of samples to light. All incubations were terminated after 120 min by addition of a volume of acetonitrile equal to the incubation volume. Control incubations were also performed in the absence of Supersomes™, in the absence of cofactor and in the absence of TQ. Samples were centrifuged (Eppendorf, Hauppauge, NY, USA; approximately 13,000*g*, 3 min, room temperature), and supernatants removed for analysis by ultra performance liquid chromatography-mass spectrometry (UPLC-MS).

### Human hepatocyte relay method

The human hepatocyte relay method used was similar to that previously reported by Di et al. [15]. Pooled cryopreserved human hepatocytes from 26 donors were obtained from Life Technologies (MA, USA). Following thawing (as per supplier guidance), hepatocytes were resuspended in Williams’ E medium (Sigma) supplemented with cell maintenance pack (Life Technologies). The cells were counted using the trypan blue exclusion method and 12-well hepatocyte plates containing 1 million cells/ml were spiked with TQ at a final concentration of 5 and 10 μM in a final incubation volume of 0.50 ml. The plates were incubated at 37 °C with 95 % oxygen/5 % carbon dioxide, 75 % relative humidity for 24 h on an orbital shaker in an incubator. At 24 h the hepatocyte suspension in the incubation plate was centrifuged (13,000*g*, 3 min, room temperature); a total of 600 µl of the supernatant was transferred to a clean 12-well plate and stored at −80 °C until the next relay experiment. For the next relay experiment, the supernatant plates were pre-warmed to 37 °C for approximately 20 min, and further hepatocytes (prepared as described previously) were added to the samples to yield a final cell density of 1 million cells/ml. The plates were incubated at 37 °C for 24 h and processed as described earlier. Six relays were undertaken to give a total incubation time of 6 days and samples were protected from light wherever possible. A drug-only control plate was run under the same conditions to investigate any non-metabolic drug breakdown. At the end of the six relay method period the entire hepatocyte suspension was collected and centrifuged (Eppendorf, Hauppauge, NY, USA) at 13,000*g* for 3 min at room temperature, and transferred to vials prior to UPLC-MS analysis.

### Tafenoquine analysis and structural identification of drug related components

Analyses of reaction phenotyping samples for TQ-related components were carried out by UPLC-MS using a Waters Acquity UPLC connected to a Waters Synapt G2S mass spectrometer using an Acquity UPLC BEH C18 column (100 × 2.1 mm, 1.7 µm; Waters Corporation, Milford, MA, USA). The mobile phase consisted of 50 mM ammonium acetate (native pH; solvent A) and acetonitrile (solvent B) at a flow rate of 0.3 ml/min. A gradient was used, starting at 5 % B then linear phase to 95 % B by 12 min, held for 1 min before returning to the initial conditions by 13.5 min with these conditions being maintained for an additional 1.5 min before subsequent injections. TQ ([M + H]^+^) was detected at *m/z* 464 using positive ion electrospray ionization. Drug-related components were identified based on charged molecular ions, mass accuracy and their collision-induced dissociation fragmentation. Authentic standards, when available, were used to compare chromatographic retention times and fragmentation patterns. Supporting data from previous studies were also used in the assignment of structures (unpublished data on file within DMPK (Drug Metabolism and Pharmacokinetics) Department, GSK).

## Results

Among 329 subjects who comprised the intent-to-treat (ITT) population in TAF112582, 237 (72 %) provided written informed consent and a blood sample for PGx research; this included 136/136 (100 %) of subjects from Peru, 98/99 (99 %) from Thailand, 3/57 (5 %) from India and 0/37 (0 %) from Brazil. Genetic data on 224 of the 237 (95 %) subjects passed standard manufacturer data quality control. Among these 224 subjects, 20 subjects were excluded from analysis as they were either lost to follow-up or treated with non-study medications with potential anti-malarial properties. In a further five subjects, metabolizer phenotype could not be unambiguously determined from *CYP2D6* genotypes, leaving a PGx analysis sample of 199 subjects. The distribution of these 199 subjects by treatment, country of origin and clinical outcome is shown in Table 1. For the post hoc PK analysis, data were available on 128 of the 134 subjects treated with TQ that were included in the PGx analysis.

**Table 1:** Clinical outcome and country by treatment in 199 subjects involved in the CYP2D6 PGx analysis

	TQ plus CQ (n = 134, total)				PQ plus CQ (n = 31)	CQ plus placebo (n = 34)
	50 mg (n = 34)	100 mg (n = 35)	300 mg (n = 37)	600 mg (n = 28)		
	N, subjects (%) per treatment arm					
Clinical outcome						
6 month relapse-free (n = 126)	18 (53)	17 (49)	32 (86)	26 (93)	23 (74)	10 (29)
Relapsed (n = 73)	16 (47)	18 (51)	5 (14)	2 (7)	8 (26)	24 (71)
Country						
India (n = 3)	1 (3)	1 (3)	1 (3)	0 (0)	0 (0)	0 (0)
Peru (n = 114)	20 (59)	21 (60)	19 (51)	16 (57)	18 (58)	20 (59)
Thailand (n = 82)	13 (38)	13 (37)	17 (46)	12 (43)	13 (42)	14 (41)

One of the 199 subjects evaluated was classified as PM (0.5 %), 38 were classified as IM (19.1 %) and 160 were classified as EM (80.4 %) (Table 2). All three subjects from India were EMs. From Peru, there was one PM (0.9 %), 12 IMs (10.5 %) and 101 EMs (88.6 %), while from Thailand, there were 26 IMs (31.7 %) and 56 EMs (68.3 %). There was a statistically significant difference (p < 0.001) in the frequency of the CYP2D6 IM phenotype between Peru (n = 12/114, 10.5 %) and Thailand (n = 26/82, 31.7 %) (see Additional file 1), but not between the four treatment arms (TQ high dose + CQ, TQ low dose + CQ, PQ + CQ, CQ only; p = 0.09) (see Additional file 2).

**Table 2:** Distribution of *CYP2D6* genotype, metabolizer phenotype and activity score

*CYP2D6* genotype	CYP2D6 metabolizer phenotype^a^	CYP2D6 activity score^b^	All subjects n = 199 N (%)	India n = 3 N (%)	Peru n = 114 N (%)	Thailand n = 82 N (%)
*4/*4	PM	0	1 (0.5)	–	1 (0.9)	–
*4/*41	IM	0.5	1 (0.5)	–	1 (0.9)	–
*1/*4	IM	1.0	11 (5.5)	–	8 (7.0)	3 (3.7)
*10/*10	IM	1.0	19 (9.5)	–	–	19 (23.2)
*10/*41	IM	1.0	2 (1)	–	–	2 (2.4)
*2/*4	IM	1.0	3 (1.5)	–	3 (2.6)	–
*41/*41	IM	1.0	2 (1)	–	–	2 (2.4)
*1/*10	EM	1.5	15 (7.5)	–	–	15 (18.3)
*1/*41	EM	1.5	6 (3)	–	3 (2.6)	3 (3.7)
*1/*9	EM	1.5	2 (1)	–	2 (1.8)	–
*2/*10	EM	1.5	14 (7)	–	–	14 (17.1)
*2/*41	EM	1.5	4 (2)	–	–	4 (4.9)
*1/*1	EM	2.0	47 (23.6)	–	35 (30.7)	12 (14.6)
*1/*2	EM	2.0	45 (22.6)	3 (100)	40 (35.1)	2 (2.4)
*2/*2	EM	2.0	27 (13.6)	–	21 (18.4)	6 (7.3)

^a^CYP2D6 poor metabolizers (PM), intermediate metabolizers (IM), extensive metabolizers (EM)

^b^CYP2D6 activity score is the sum of the per-allele scores; a null allele having a score of 0, a deficient allele a score of 0.5 and a normal allele a score of 1 [12]

**Fig. 1:**
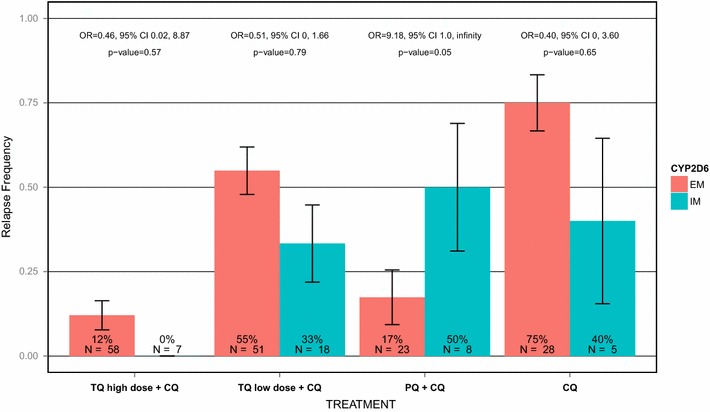
Clinical outcome by treatment and CYP2D6 status. Relapse frequency is the percentage (%) of subjects who experienced a relapse, with *error bars* representing the standard error of the mean. Odds ratios (ORs) and 95 % confidence intervals (95 % CI) were from analyses comparing the relapse frequency of reduced metabolizers (IMs) with that of extensive metabolizers (EMs) within each treatment group. Exact logistic regression was used to derive ORs, except for the TQ high dose arm (TQ 300 mg/TQ 600 mg), in which Fisher’s Exact test was used with a correction of 0.05 to each cell as there were no IM subjects who relapsed. One-sided p values (p) are displayed (see “Methods” section)

No evidence of association between CYP2D6 IM phenotype and increased frequency of clinical relapse of *P. vivax* infection was seen in either of the TQ treatment groups using a one-sided test as described in the Methods (Fig. 1). Relapse frequencies were actually lower in IM subjects in both TQ treatment groups, a trend that was also observed in the CQ only arm. Notably, all seven IM subjects in the TQ high dose (300/600 mg) treatment group remained relapse-free during the 6 months of follow-up. By contrast, and in support of previous findings [5, 7], the relapse frequency was higher in PQ-treated subjects who were IM (50 %) than in EM subjects (17 %), p = 0.05, odds ratio = 9.18 (95 % CI 1.00, ∞). The single PM subject in the clinical study was randomized to the CQ arm and relapsed. Comparable results were obtained using the CYP2D6 AS approach: no effect was observed in the TQ arms or in the CQ arm, whether the PM subject was included in the CQ arm or not, while a non-significant trend for lower AS scores in subjects who relapsed was observed in the PQ arm (p = 0.06).

**Fig. 2:**
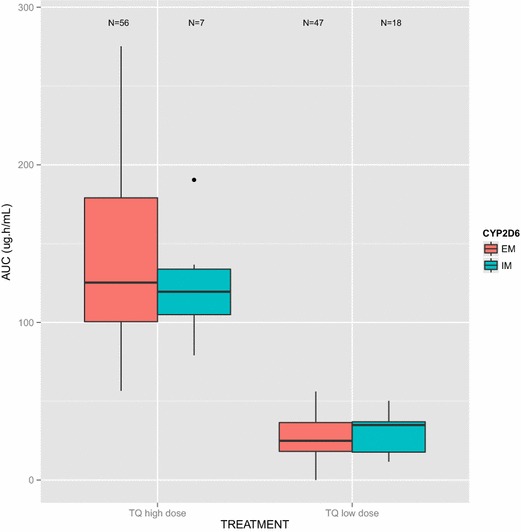
*Box whisker plot* of TQ AUC by CYP2D6 status. The *box whisker plots* are displayed separately for the TQ high dose arm (TQ 300 mg/TQ 600 mg) and TQ low dose (TQ 50 mg/TQ 100 mg) arms, respectively. The median AUC is represented by the *thick horizontal line*, while the *lower* and *upper box edges* (‘hinges’) represent the quartiles (the 25th and 75th percentiles). The interquartile range (IQR) is the distance between the first and third quartiles. The upper (lower) whisker extends from the upper (lower) hinge to the highest (lowest) value that is within 1.5 *IQR of the hinge

CYP2D6 phenotype had no significant effect on TQ AUC in either the TQ high dose (p = 0.72) or TQ low dose (p = 0.24) groups (Fig. 2). The median AUC (interquartile range) in the TQ high dose group was 125.26 μg.h/ml (100.34–179.65 μg.h/ml) in EM subjects and 119.52 μg.h/ml (93.17–136.67 μg.h/ml) in IM subjects. In the TQ low dose group, the median AUC (interquartile range) was 24.88 μg.h/ml (17.86–36.50 μg.h/ml) in EM subjects and 34.92 μg.h/ml (16.89–37.04 μg.h/ml) in IM subjects.

**Fig. 3:**
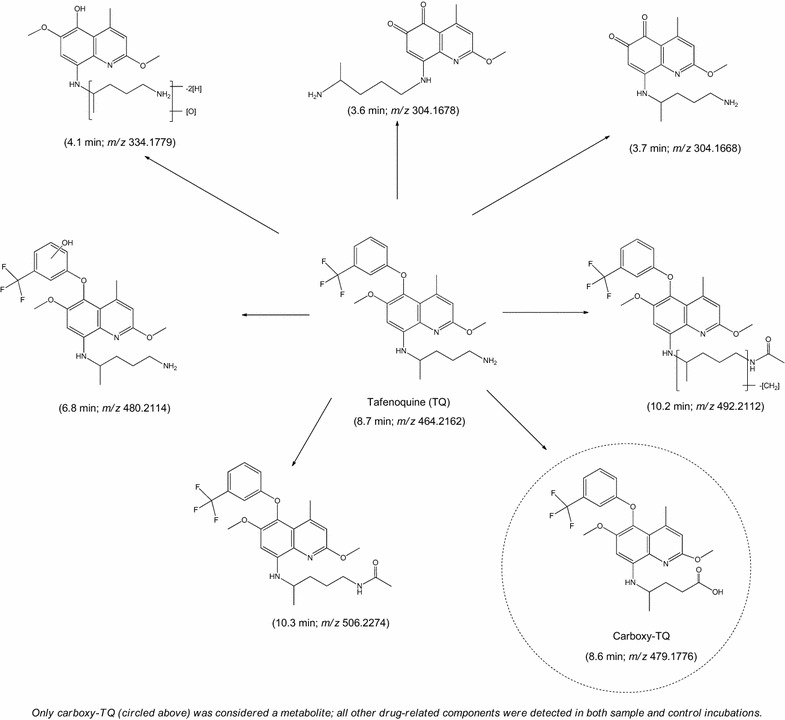
Summary of TQ-related components detected in incubations of TQ with recombinant human CYP Supersomes. TQ-related compounds detected in 5 and 10 μM incubations of TQ with recombinant human CYP enzymes (Supersomes™), hepatocytes and appropriate controls (retention time and protonated molecular ion observed by UPLC-MS in *parenthesis*). The Carboxy-TQ derivative observed after prolonged incubation is *circled*

*In vitro* metabolism of TQ studied in CYP Supersomes™ and human hepatocytes indicated minimal metabolism of TQ. A summary of the structural information for the drug-related components of TQ are shown in Fig. 3, however, it should be noted that the majority of these components were observed in both sample and control incubations at comparable levels and are therefore likely to be due to degradation of TQ, and not true metabolites. In all incubations (including controls), the prominent component was TQ, with additional components resulting from O-demethylation, O-dearylation, oxidation, and N-acetylation. Following 120-min incubations of 5 and 10 µM TQ with Supersomes™ containing overexpressed CYP1A2, CYP2C8, CYP2C9, CYP2C19, CYP2D6, or CYP3A4, no additional drug-related components were detected which were not observed in the control incubations, indicating no or negligible metabolism of TQ had occurred. Following a six-day human hepatocyte relay, a minor component, carboxy-TQ, was the only additional TQ-related component detected by UPLC-MS. Carboxy-TQ was not observed in control incubations, indicating that minimal metabolism had occurred even after this long incubation period and despite a good metabolic capacity of the preparation (viability of the hepatocytes used ranged from 91–93 %).

## Discussion

PQ has been approved for treating relapsing malaria since its discovery in the 1950s [16], however, it is not widely used in areas where *P. vivax* is endemic [1]. Factors that have hampered PQ’s effectiveness in developing countries include poor compliance, due to a lengthy treatment regime (typically 14 days), and concerns around the risk of haemolysis in G6PD-deficient individuals. While both PQ and TQ have G6PD liability, TQ’s long half-life has enabled a single dose regime in current clinical trials, which could be expected to result in good patient compliance.

As highlighted recently, a potential CYP2D6 PGx liability for 8-AQs would represent a significant obstacle to large-scale prophylaxis and eradication campaigns for *P. vivax* [8]. Here, a retrospective PGx analysis was performed on the effect of inferred CYP2D6 phenotype on TQ PK and TQ relapse efficacy in participants from TAF112582, a published randomized phase 2b clinical trial [2]. Given the known global variation in allele frequencies, the difference in CYP2D6 metabolizer phenotype observed between subjects from Thailand and Peru was as expected and driven primarily by CYP2D6*10, a functionally-deficient allele common in Asian countries [17]. These results demonstrate that TQ efficacy is not diminished in *P. vivax*-infected individuals who have reduced CYP2D6 metabolism nor is TQ PK affected by CYP2D6, a finding that is at odds with previously published research in mice [8, 9]. However, these data support the growing body of evidence implicating reduced CYP2D6 metabolism in PQ failure [3–7].

In contrast to PQ [4], metabolites of TQ, in particular those potentially resulting from CYP2D6 metabolism, are considered less likely to influence the efficacy of this drug, which agrees with findings from the PGx and PK analyses conducted in this report. At present, the mechanism of action for TQ is not known as few metabolites have been observed in subjects dosed with TQ. Further support for a differential effect of CYP2D6 on TQ and PQ metabolism comes from various in vitro human liver drug metabolism investigations (including those described within this publication), all of which have demonstrated minimal hepatic metabolic turnover of TQ. This would suggest that unlike PQ, metabolism of TQ may not be an important factor in vivo for efficacy against *P. vivax*. Unpublished analyses for TQ metabolites in plasma (and blood) collected from healthy female volunteers dosed orally with TQ (up to 300 mg) for 3 days are consistent with in vitro findings reported here. The only circulating oxidative metabolite of TQ observed in 0–144-hour pools was carboxy-TQ, which was estimated by ultraviolet spectroscopy and mass spectrometry to be at very low levels compared to unchanged TQ (unpublished data on file within DMPK Department, GSK).

Taken together, the in vivo and in vitro data presented here provide evidence that TQ efficacy against *P. vivax* might not be reliant on CYP2D6 metabolism to the same extent as PQ. As indicated previously, the translation of genotype information into metabolizer phenotype is challenging and small sample size limits conclusions that can be drawn from the PGx component of this study, including whether TQ efficacy is different in CYP2D6 PM and IMs. Therefore, the current hypothesis and any potential CYP2D6 effects on TQ and PQ efficacy will be further evaluated in the ongoing phase 3 programmes (TAF112582 Part 2 and TAF116564).

## Conclusion

Converging evidence suggests that TQ *P. vivax* radical cure efficacy in humans is not impacted in individuals with decreased CYP2D6 metabolism to the same extent as PQ.

## Additional files

10.1186/s12936-016-1145-5 The percentage of intermediate metabolizers (IM) by country. The percentage of subjects who were intermediate metabolizers (IM) is shown with error bars representing the standard error of the mean. The three subjects from India are not shown as all were extensive metabolizers (EMs).
10.1186/s12936-016-1145-5 The percentage of intermediate metabolizers by treatment. The percentage of subjects who were intermediate metabolizers (IM) with error bars representing the standard error of the mean.

## Abbreviations

8-AQ: 8-aminoquinoline
AS: CYP2D6 activity score
AUC: area under the curve
CQ: chloroquine
CYP: cytochrome P450
DMPK: drug metabolism and pharmacokinetics
EM: extensive metabolizer
FDA: federal drug administration
G6P: glucose-6-phosphate
G6PD: glucose-6-phosphate dehydrogenase
GSK: GlaxoSmithKline
HPLC: high-performance liquid chromatography
IM: intermediate metabolizer
ITT: intent to treat
MMV: medicines for malaria venture
NADP: β-nicotinamide adenine dinucleotide phosphate
PGx: pharmacogenetics
PM: poor metabolizer
PQ: primaquine
TQ: tafenoquine
UPLC-MS: ultra performance liquid chromatography-mass spectrometry
w/v: weight per volume
