# Supplementary figures and images for: Tafenoquine treatment of Plasmodium vivax malaria: suggestive evidence that CYP2D6 reduced metabolism is not associated with relapse in the Phase 2b DETECTIVE trial
Source: Malar J. 2016 Feb 18;15:97. doi: 10.1186/s12936-016-1145-5 (PMC4757974; doi:10.1186/s12936-016-1145-5)

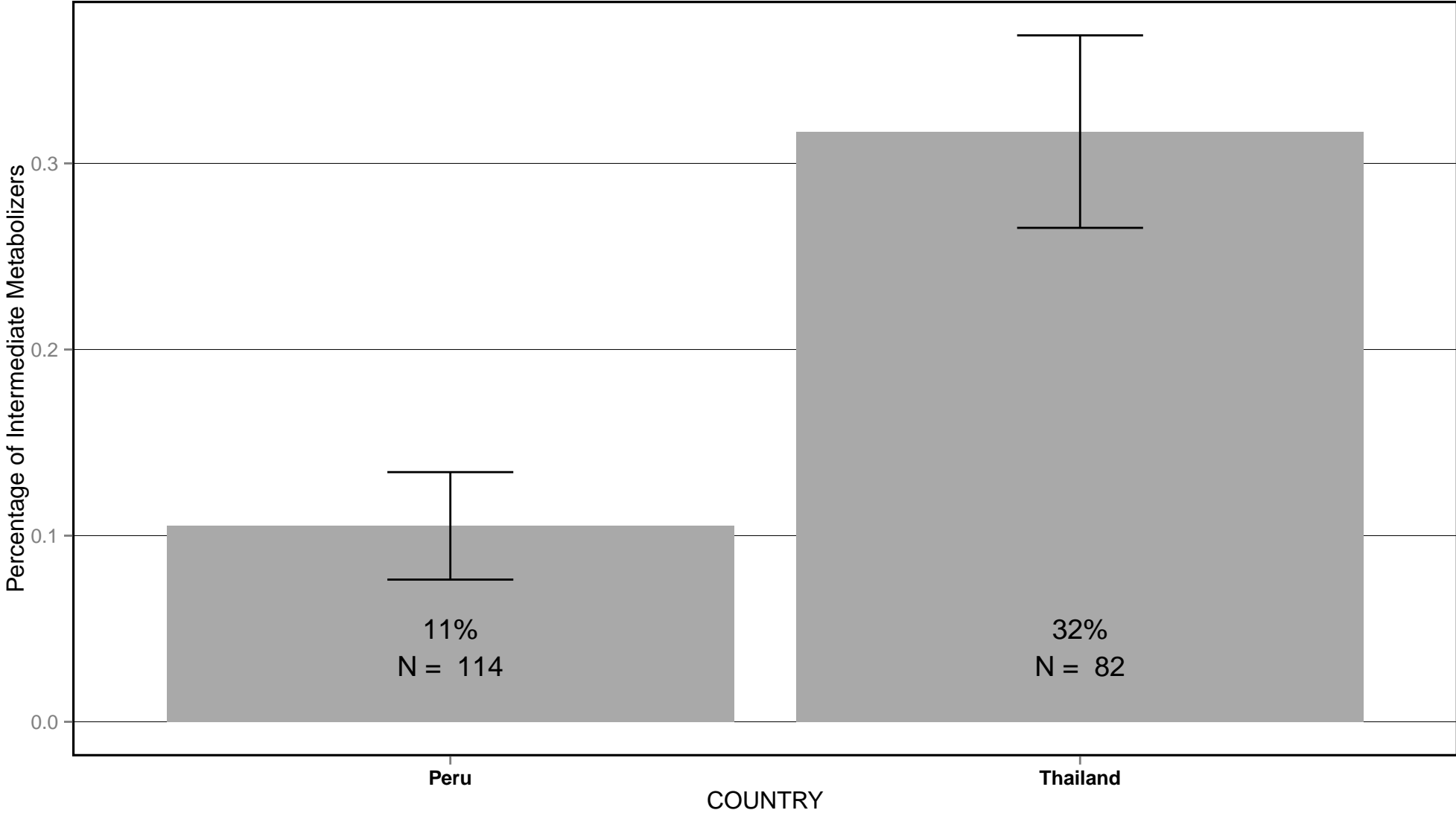

Supplement: Supplementary file 1 — 10.1186/s12936-016-1145-5 The percentage of intermediate metabolizers (IM) by country. The percentage of subjects who were intermediate metabolizers (IM) is shown with error bars representing the standard error of the mean. The three subjects from India are not shown as all were extensive metabolizers (EMs). [file 12936_2016_1145_MOESM1_ESM.pdf]

Percentage of Intermediate Metabolizers

0.3

0.2

0.1

0.0

TQ high dose + CQ

TQ low dose + CQ

PQ + CQ

CQ

TREATMENT

11%  
N = 65

26%  
N = 69

26%  
N = 31

15%  
N = 34

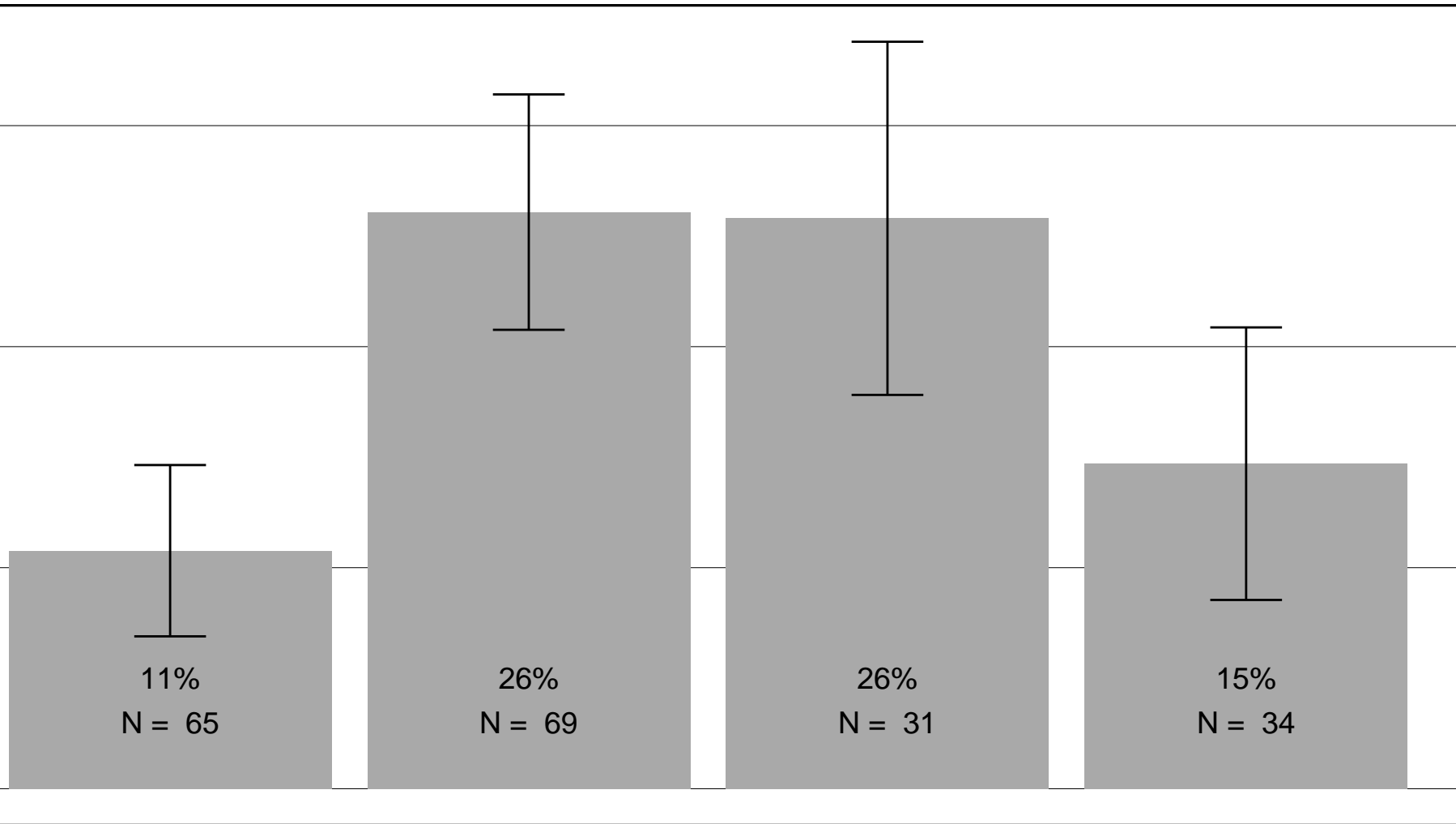

Supplement: Supplementary file 2 — 10.1186/s12936-016-1145-5 The percentage of intermediate metabolizers by treatment. The percentage of subjects who were intermediate metabolizers (IM) with error bars representing the standard error of the mean. [file 12936_2016_1145_MOESM2_ESM.pdf]
